# Identification of Socio-demographic and Psychological Factors Affecting Women’s Propensity to Breastfeed: An Italian Cohort

**DOI:** 10.3389/fpsyg.2016.01872

**Published:** 2016-11-29

**Authors:** Valentina E. Di Mattei, Letizia Carnelli, Martina Bernardi, Chiara Jongerius, Chiara Brombin, Federica Cugnata, Anna Ogliari, Stefania Rinaldi, Massimo Candiani, Lucio Sarno

**Affiliations:** ^1^Faculty of Psychology, Vita-Salute San Raffaele UniversityMilan, Italy; ^2^Clinical and Health Psychology Unit, Department of Clinical Neurosciences, IRCCS San Raffaele HospitalMilan, Italy; ^3^Faculty of Social and Behavioural Sciences, Leiden UniversityLeiden, Netherlands; ^4^University Centre of Statistics in the Biomedical Sciences, Vita-Salute San Raffaele UniversityMilan, Italy; ^5^Department of Obstetrics and Gynecology, IRCCS San Raffaele HospitalMilan, Italy; ^6^Faculty of Medicine, Vita-Salute San Raffaele UniversityMilan, Italy

**Keywords:** breastfeeding, neuroticism, breastfeeding intention, psychological factors, Italy

## Abstract

**Background:** Exclusive breastfeeding until 6 months postpartum is a World Health Organization objective and benefits have been demonstrated for both mother and infant. It is important to clarify which factors influence breastfeeding intentions. Our objective was to assess and identify socio-demographic and psychological factors associated with breastfeeding intention in a sample of pregnant Italian women.

**Materials and Methods:** This prospective study included 160 pregnant women. The following psychological constructs were measured using standardized questionnaires: anxiety, prenatal attachment, adult attachment, personality traits, and intention to breastfeed. Socio-demographic data were also collected using a self-report questionnaire. Assessment took place after the 20th gestational week.

**Results:** Self-employment, age and feeding received as an infant were significantly related to breastfeeding intention. Regarding psychological factors, we also found that Neuroticism was negatively associated with mother’s breastfeeding intentions. Relationships between psychological constructs and breastfeeding attitude were examined and represented within a graphical modeling framework.

**Conclusion:** It may be possible to identify women that are less inclined to breastfeed early on in pregnancy. This may aid healthcare staff to pay particular attention to women who show certain socio-demographic and psychological characteristics, so as to fulfill more focused programs.

## Introduction

Encouraging mothers to breastfeed is an important World Health Organization (WHO) objective and a primary health promotion strategy. The World Health Organization [WHO], 2001 and the Italian Ministry of Health, 2007 recommend exclusive breastfeeding for at least 6 months and continuation of breastfeeding together with other food until 2 years of age.

The WHO has reported well-established short-term infant benefits of breastfeeding, especially the reduction of mortality and morbidity from infectious diseases (World Health Organization [WHO], 2000). Furthermore, the WHO conducted a systematic review and meta-analysis on the consequences of breastfeeding and found that there are long-term benefits to breastfeeding too; for example, there is strong evidence of an effect of breastfeeding on IQ (Horta and Victora, 2013). Benefits have also been reported for the mother: in the long-term, mothers who breastfeed tend to be at lower risk of premenopausal breast cancer and ovarian cancer (Leon-Cava et al., 2002). There is also some evidence that by breastfeeding mothers may become calmer and more sociable, which in turn, may promote attachment security (Jansen et al., 2008).

Despite these well-documented advantages and preventive benefits of breastfeeding, few women worldwide meet the WHO’s recommendation of exclusive breastfeeding up to six months postpartum (de Jager et al., 2014); breastfeeding rates tend to decrease dramatically within the first weeks after-birth (World Health Organization [WHO], 2014). In Italy specifically, the Health Ministry has recently promoted breastfeeding practices and has urged hospitals to adhere to the Baby Friendly Hospital Initiative (World Health Organization [WHO], 2014; UNICEF, 2015) in an attempt to improve maternity services (Istituto Nazionale Della Previdenza Sociale, 2016). Despite these efforts, the current Italian breastfeeding rates are as follows: around 90% of mothers breastfeed immediately after birth, 77% at hospital discharge, 31 and 10% at 4 and 6 months after pregnancy, respectively (Davanzo and De Cunto, 2013). Furthermore, there seems to be an imbalance in the way these rates are distributed in Italy; breastfeeding rates are higher in the North of Italy compared to the South. This may be due to the better health and welfare services offered in the North of the country, but it has also recently been determined that other social and personal determinants may affect these rates, such as individual attitudes (Romero et al., 2006). Moreover, in Italy there is no monitoring and periodic validation system for infant feeding practices. For this reason, a recent Position Statement has been developed to promote breastfeeding practices in Italy (Davanzo et al., 2015). Since knowledge that breastfeeding is the optimal method of infant feeding does not seem to be enough to encourage women to breastfeed exclusively (Gill et al., 2007), investigating which factors are associated to mothers’ intention to breastfeed could help encourage mothers to breastfeed and aid them in sustaining this option for longer periods of time.

Maternal psychological variables have been shown to influence breastfeeding and can be seen as potential intervention targets (O’Brien et al., 2008). Studies have found that anxiety (O’Brien et al., 2008) and breastfeeding intentions (de Jager et al., 2014) were predictive of breastfeeding duration. Furthermore, women’s attitudes regarding breastfeeding (Scott et al., 2006), adult attachment (Akman et al., 2008), and pre-natal attachment (Scharfe, 2012) have been shown to be associated with choice and duration of breastfeeding.

Despite the established association between psychological maternal characteristics and breastfeeding duration, there has been little empirical examination of the role of maternal personality on infant feeding decisions (Brown, 2014). According to the Five-factor model (FFM) (McCrae and Costa, 1987), personality can be conceptualized in five basic dimensions: Extraversion, Agreeableness, Conscientiousness, Neuroticism, and Openness to experience. Of these traits, Neuroticism, Extraversion, and Conscientiousness are most often related to breastfeeding initiation and continuation (Bick and Chang, 2014; Brown, 2014).

Furthermore, socio-demographic factors have also been associated with maternal intention to breastfeed. These include older age (Lutsiv et al., 2013; Biro et al., 2014), primiparity (Huang et al., 2004; Britten et al., 2011; Lutsiv et al., 2013), higher maternal education (Riva et al., 1999; Huang et al., 2004), and employment (Attanasio et al., 2013).

Studies that have analyzed breastfeeding practices in the past have either considered the behavioral choice to breastfeed (Davanzo and De Cunto, 2013) or the propensity to breastfeed (Lutsiv et al., 2013). Even though breastfeeding intentions do not equate to actual breastfeeding rates because a mother can encounter various difficulties during weaning, maternal propensity to breastfeed has been shown to correlate with the choice and duration of breastfeeding (Lutsiv et al., 2013). For this reason, measuring maternal intentions to breastfeed is useful as it can predict how a mother will feed her child after labor.

In light of these considerations, the aim of our study was to determine which factors have an impact on Italian women’s propensity to breastfeed. Socio-demographic (e.g., women’s age, education, occupation, marital status, and parity) and psychological factors, namely anxiety (O’Brien et al., 2008), prenatal attachment (Huang et al., 2004), adult attachment (Akman et al., 2008), and personality traits (Bick and Chang, 2014; Brown, 2014) were investigated. We hypothesized that there would be specific socio-demographic and psychological characteristics distinguishing mother’s who intended to bottle-feed from those who intended to breastfeed. By identifying factors involved in breastfeeding intention, we hope to aid mothers and healthcare providers in promoting breastfeeding and targeting mothers who are at risk of not breastfeeding. The lack of Italian research in breastfeeding practices served as an incentive to investigate what factors influence pregnant Italian women to breastfeed or not. Moreover, Romero et al. (2006) have emphasized that more information is needed for health professionals with regards to lactation in Italy: this study aimed to answer to this need.

## Materials and Methods

### Sample Selection and Recruitment

Pregnant women who were attending routine antenatal appointments at a University Hospital in Milan, Italy, were invited to take part in the study. Eligible women had to be over 18, Italian, with at least an elementary school certificate, expecting a singleton or twin, and agreed voluntarily to take part in the research. All women were from the North of Italy. Moreover, they had to be at least in their 20th week of pregnancy; this criterion was crucial for the measurement of prenatal attachment as only after the 20th week can a mother start to perceive fetal movements (Royal College of Obstetricians and Gynaecologists, 2011). Following these criteria, 160 women took part in the study (*n* = 160). Data collection began in January, 2013 and concluded in December, 2014. The study was approved by the San Raffaele Hospital Medical Ethical Committee; a written informed consent was obtained from all the participants at the time of questionnaire completion.

### Measures and Procedure

The study was conducted after one of the antenatal visits of the third trimester of pregnancy. Demographic and obstetric information were collected via a self-report questionnaire including: date of birth, level of education (lower secondary education or less, upper secondary education, and higher education), employment status (employed, self-employed, and unemployed), mother’s decision about going back to work, marital status, parity (primipara or pluripara), feeding received by the mother as a baby (breastfeeding, formula feeding or mixed), gestational age (less or more than 32 weeks), history of miscarriage or stillbirth (binary variable) and current or past psychiatric disorders.

Validated questionnaires were administered to assess psychological variables. The first questionnaire was the *State-Trait Anxiety Inventory* (STAI) (Spielberger et al., 1983), a widely used measure of anxiety. It consists of two subscales: the state and trait subscales. Responses are given on a 4-point Likert scale; for the state scale scores range from 1 “not at all” to 4 “very much so” and for the trait scale from 1 “almost never” to 4 “almost always”. Total scores range from 20 to 80 for each subscale. Scores are grouped into three categories: low anxiety (scores of 20-39), medium anxiety (scores of 40-59) and high anxiety (scores of 60-80). In this study we used the Italian version of the questionnaire (Pedrabissi and Santinello, 1996) where both the state scale (Cronbach’s alpha coefficient α ranging from 0.91 to 0.95) and the trait scale (α range = 0.85-0.90) showed excellent internal consistency reliability.

The *Prenatal Attachment Inventory* (PAI) (Muller, 1993) was used to assess the extent of the “unique and affectionate relationship that develops between a mother and her unborn baby”^31^. It consists of 21 Likert-type items, scored on a four-point scale, ranging from 1 “almost always” to 4 “almost never”. A total score (from 21 to 84) is obtained by summing the 21 items, with higher scores indicating a greater maternal-fetal attachment. The Italian version of the PAI was administered; this version has shown good reliability (α = 0.869) and high construct validity (Della Vedova et al., 2008).

The *Attachment Style Questionnaire* (ASQ) (Feeney et al., 2004) is a self-report questionnaire designed to measure adult attachment. It consists of 40 Likert scale items; each item is rated on a 6-point scale, ranging from 1 “totally disagree” to 6 “totally agree”. The items are assigned to five scales: Discomfort with Closeness (10 items, range 10-60), Need for Approval (7 items, range 7-42), Preoccupation with Relationships (8 items, range 8-48), Relationships as Secondary (7 items, range 7-42) and Confidence (8 items, range 8-48) (Feeney et al., 2004; Fossati et al., 2003). The ASQ shows adequate internal consistency, with Cronbach’s alpha coefficients ranging from 0.64 to 0.78 in the Italian version of this questionnaire used in the present study (Fossati et al., 2003).

The *Big Five Inventory* (BFI) (John et al., 1991) is a self-report questionnaire designed to assess the traits defined by the Five-Factor Theory of Personality. The five factors include: Conscientiousness (orderliness, responsibility and dependability; 9 items, range 9–45), Agreeableness (kindness, cooperativeness and trust; 9 items, range 9–45), Extraversion (talkative, energetic and assertive; 8 items, range 8–40), Neuroticism (tense, moody and anxious; 8 items, range 8–40 points) and Openness (originality, curiosity, and ingenuity; 10 items, range 10–50). It consists of 44 items, which are rated on a five-point Likert scale, from 1 “disagree a lot” to 5 “agree a lot”. In this study the Italian version (Ubbiali et al., 2013) of the BFI was used; the internal consistency range was α = 0.69-0.84.

All women completed the *Iowa Infant Feeding Attitude Scale* (IIFAS) (De la Mora et al., 1999). The IIFAS was used to assess parents’ intention and attitudes toward infant feeding. It consists of 17 attitude questions on a 5-point Likert scale ranging from 5 “strongly agree” to 1 “strongly disagree”. Questions were divided so that half were favorable to breastfeeding and the other half to formula feeding. Items that favored formula feeding were reverse scored and a total score was obtained. IIFAS scores could range from 17 to 85, with higher scores indicating a more positive attitude toward breastfeeding. IIFAS scores of 65 or above indicate that women are likely to breastfeed (De la Mora et al., 1999). Scores of 50 or less indicate a positive attitude toward infant formula feeding (Sittlington et al., 2007). Wilkins et al. (2012) added a third group of scores ranging between 51-64 that identified women who did not have a firm preference for breastfeeding or formula feeding. The IIFAS appears to be very reliable (De la Mora et al., 1999), with Cronbach’s alpha ranging from 0.85 to 0.86. In this study a translated version of the IIFAS, approved by the original authors, was used.

### Statistical Analysis

Descriptive statistics of psychometric data and other continuous variables were presented as mean, standard deviation and range, while for categorical variables frequencies were reported. Student’s *t*-test or ANOVA were used to test differences in the mean IIFAS score across demographic and obstetric groups. The Pearson correlation coefficient was used to assess the strength of the relationships between psychological variables and the IIFAS score. In addition, a multiple linear regression model was fit to identify predictors of IIFAS score. Backward stepwise procedures were applied to select the most parsimonious model. Analyses were performed using R statistical software (R Development Core Team, 2011) and the significance threshold was set at 0.05.

Graphical models were used to analyze relationships between IIFAS and psychometric scales collected in the study (Hojsgaard et al., 2012). Since they combine graph and probability theory, these models represent a flexible tool that provides a powerful framework for decision-making purposes. In graphical models, each random variable is associated with a node in a graph and edges represent conditional dependency between variables. Whenever links are missing, variables are conditionally independent given the remaining variables. gRapHD package in R (Abreu et al., 2010), that applies the Chow-Liu algorithm (Chow and Liu, 1968), was used to derive the final graphical models.

## Results

### Sample Description

The average age of the mothers in this study was 33.92 years (*SD* = 4.38), ranging from 19 to 45 years. In **Table 1**, mother’s demographic and obstetric characteristics are shown. Most women were at least in their 32nd gestational week (68.75%). Around 39% of our sample had a higher, post-secondary education. The majority of participants were employed (75.62%) and married (75%). About 86% of women were having their first child and 51% had been breastfed as infants.

**Table 1:** Demographic and obstetric characteristic and IIFAS scores.

Characteristic	Frequency	%	IIFAS score	
			*Mean* (*SD*)	*p*-value
Level of education				
Lower secondary education or less	29	18.13%	62.31 (8.52)	0.064^§^
Upper secondary education	68	42.5%	66.21 (7.70)	
Higher education	63	39.38%	65.63 (6.98)	
Employment status				
Employee/Unemployed/student	127	79.38%	64.52 (7.94)	0.004^†^
Self employed	33	20.62%	68.18 (5.73)	
Marital status				
No married	40	25%	64.45 (8.14)	0.454^†^
Married	120	75%	65.55 (7.52)	
Parity				
Primiparas	137	85.62%	65.62 (7.34)	0.250^†^
Multiparas	23	14.37%	63.217 (9.32)	
Feeding received by mother as an infant				
Breastfeeding/Mixed	87	54.38%	67.66 (5.87)	< 0.001^†^
Formula feeding	73	45.62%	62.44 (8.58)	
Miscarriage or stillbirth				
Yes	27	16.88%	65.43 (7.25)	0.644^†^
No	133	83.13%	64.52 (9.59)	
Return to work (months)				
0-3	12	8.28%	66.33 (9.43)	0.652^§^
4-6	41	28.28%	64.61 (8.24)	
7-12	71	48.97%	66.01 (6.64)	
>12	21	14.48%	64.14 (8.60)	
Gestational age (weeks)				
26-32	50	31.25%	65.94 (8.07)	0.475^†^
>32	110	68.75%	64.97 (7.50)	

*^†^*p*-value *t*-test.*

*^§^*p*-value ANOVA.*

The majority of women declared to plan to return to work in the next 7-12 months (49%) and around 17% of them had previously experienced a miscarriage or stillbirth. The average score on the IIFAS for the overall sample was 65.28 (*SD* = 7.67, ranging from 45 to 85). When assessing differences in mean IIFAS scores between groups, determined on the basis of in demographic/obstetric characteristics, we found that attitude towards breastfeeding significantly differs depending on the mother’s employment (*p* = 0.004, comparison between self employed and all the other categories, e.g., employed/unemployed/student) and the feeding she received as a child (*p* < 0.001).

In **Table 2**, descriptive statistics of psychometric variables were reported along with their correlations with IIFAS. We found that only Neuroticism (ρ = -0.214, *p* = 0.006) was negatively and significantly associated with mothers’ feeding intention.

**Table 2:** Descriptive statistics of psychometric data and correlations with IIFAS total score.

	*Mean* (*SD*)	Range (min-max)	Correlations with IIFAS score	
			*r* Pearson	*p*-value
State-Trait Anxiety Inventory				
State	36.27 (10.26)	20**-**72	**-**0.10	0.191
Trait	37.88 (8.11)	20**-**64	**-**0.12	0.134
Prenatal Attachment Inventory				
PAI	61.09 (9.13)	29**-**79	**-**0.02	0.820
Attachment Style Questionnaire				
Discomfort with Closeness	34.20 (5.44)	20**-**58	**-**0.07	0.396
Need for Approval	18.93 (5.11)	8**-**37	**-**0.01	0.918
Preoccupation with Relationship	26.91 (4.84)	14**-**40	**-**0.03	0.683
Relationships as Secondary	13.96 (4.17)	7**-**26	**-**0.10	0.193
Confidence	32.74 (3.85)	21**-**43	0.01	0.871
Big Five Inventory				
Conscientiousness	35.76 (5.08)	17**-**45	0.11	0.153
Agreeableness	33.98 (4.49)	21**-**44	0.11	0.176
Extraversion	26.54 (5.82)	10**-**38	0.07	0.418
Neuroticism	23.14 (6.00)	8**-**38	**-**0.21	0.006
Openness	36.78 (6.48)	15**-**50	0.09	0.250

### Multivariate Analyses

From the multivariate analysis significant roles of age (*p* = 0.025) and being self-employed (*p* = 0.019) emerged as promoters of breastfeeding attitudes (older and self-employed women scored higher on the IIFAS), while Neuroticism (*p* = 0.014), having been formula-fed as a child (*p* < 0.001) and already having children (*p* = 0.042) significantly reduced the IIFAS score (see **Table 3**).

**Table 3:** Estimated regression model selected by backward stepwise procedure.

	*B*	*p*-value
Intercept	63.01	<0.001
Age	0.29	0.026
Self employed	3.26	0.019
Multiparas	**-**3.23	0.042
Formula feeding	**-**5.15	<0.001
Neuroticism	**-**0.23	0.015

Graphical models were used to visualize connections between breastfeeding attitude and psychometric data. This allowed us to highlight a link between IIFAS total score and Neuroticism, confirming the previous result obtained in the multiple regression model, and between Neuroticism and trait anxiety. Moreover, the model emphasized relations between attachment styles and anxiety.

## Discussion

Breastfeeding intentions seem to be affected by a number of socio-demographic and psychological factors. On the basis of the thresholds provided in the literature (De la Mora et al., 1999) IIFAS scores obtained by women in our study were indicative of intention to breastfeed.

In particular, older mothers had a significantly higher IIFAS score (positive sign of age coefficient in the regression model). A recent study revealed that while younger women were as likely to initiate breastfeeding as older women, they had almost twice the odds of not breastfeeding at 6 months (Biro et al., 2014). In another study it was found that the odds of intending to breastfeed were higher amongst older women (Lutsiv et al., 2013). Other studies have shown similar results (Dodgson et al., 2003; Britten et al., 2011), where older age had positive effects on the intention to breastfeed. Older women could be more confident and have more life experience and knowledge regarding breastfeeding and thus have a higher propensity to breastfeed (Brand et al., 2011).

Regarding employment, we found significant differences between different employment categories. A relationship between breastfeeding and employment has been shown (Attanasio et al., 2013). Full-time maternal employment has been associated with early cessation of breastfeeding. Intention to return to work and full-time postpartum employment are associated with non-initiation of breastfeeding (Hawkins et al., 2007). Specifically, we found that self-employed women (e.g., free-lance lawyers, business consultants and journalists) were more inclined to breastfeed. Skafida (2012) also found that mothers who were part-time self-employed were more likely to exclusively breastfeed for six or more weeks, when compared to unemployed or full-time working mothers. Skafida (2012) suggests that self-employed mothers can combine work-related tasks and breastfeeding more easily as they are likely to work from home. Our study supports this finding.

Our results support existing knowledge (Huang et al., 2004; Lutsiv et al., 2013) on intention to breastfeed by confirming its association with parity. Multiparas had less intention to breastfeed. Although this has been found in the literature, no study, to our knowledge, has investigated the underlying cause for this association. We hypothesize that this result could be related to the quality of previous experiences with infant feeding (e.g., a previous negative experience with breastfeeding could influence future feeding intention) or to a different family environment (e.g., due to the presence of other children).

We also found that the type of feeding received by the mother when she was young was statistically significant. Women who were breastfed as children were more likely to breastfeed their children compared to formula-fed mothers. This is in line with previous research that found that breastfeeding mothers were more likely to have been breastfed themselves (Meyerink and Marquis, 2002). Regardless of socio-demographic differences mothers’ prior personal experiences had an impact on their breastfeeding practices. The knowledge of having been breastfed could bring a degree of familiarity with breastfeeding that mothers who were formula-fed do not have (Meyerink and Marquis, 2002).

Socio-demographic variables are often more difficult to modify, offering little opportunity to alter breastfeeding choice (O’Brien et al., 2008), however, they may be helpful in identifying women who are at risk of early weaning and in implementing programs that can increase breastfeeding duration.

With regards to psychological maternal variables, we found that Neuroticism was associated with intention to breastfeed. The personality trait of Neuroticism is characterized by anxiety, fear, worrying, frustration, and loneliness (Eysenck and Eysenck, 1985). The higher a pregnant woman scored on the Neuroticism scale of the Big Five questionnaire, the less her intention to breastfeed. This result has been found previously in the literature; Newton (1971) found that mothers who breastfed displayed significantly less Neuroticism than those who did not. Alder and Bancroft (1988) found that women who persisted in breastfeeding were less neurotic on the Eysenck Personality Inventory. Other studies have indicated that high scores in Neuroticism could be associated to antenatal depression, which could in turn affect breastfeeding intentions (O’Neill et al., 1990). Thus a mother’s personality could vary the choice of feeding (Newton, 1971).

To better understand the connections between the psychometric variables measured in our study, we explored the underlying structure of the data using a graphical model, which confirmed a connection between the IIFAS Total score and Neuroticism. Moreover, from this model, a connection between Neuroticism and anxiety emerged, specifically trait anxiety. Neuroticism has been strongly associated with anxiety (Widiger and Trull, 1992), and individuals characterized with high Neuroticism tend to worry often, thus in turn enhancing anxiety symptoms (Muris et al., 2005). There is evidence in the literature that anxiety, Neuroticism and worrying are linked (Roelofs et al., 2008). Moreover, Neuroticism is strongly related to: Axis I psychopathology (e.g., mood and anxiety disorders), low subjective well-being and physical health problems. Neurotic individuals respond more poorly to environmental stressors and minor frustrations are hopelessly difficult. These individuals tend to respond with worry, anticipatory anxiety and pessimism (Ritter and Lampkin, 2011). It is possible that an underlying anxious tendency could influence neurotic individuals to be less inclined to satisfy others’ needs and therefore less inclined towards breastfeeding their new-born children.

**FIGURE 1:**
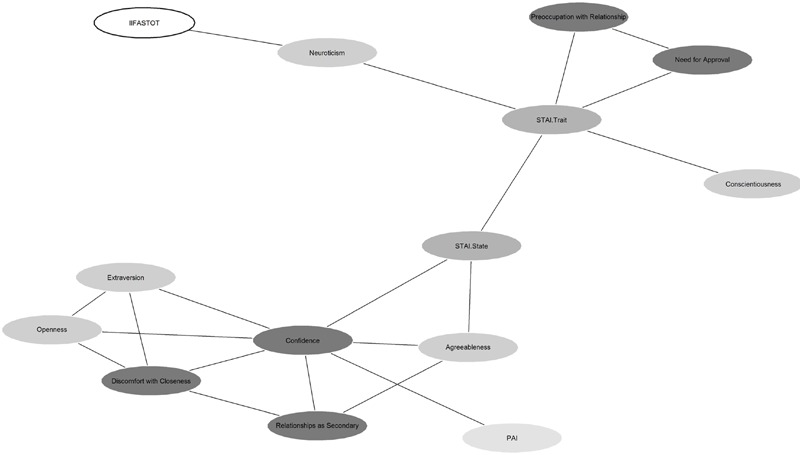
**Graphical model showing underlying relationships between IIFAS and psychometric scales.** Different shades of gray have been used to highlight groupings of psychometric scales derived from the same questionnaire (from light gray for the PAI scales to dark gray for the ASQ scales). For the outcome variable a white ellipse with a thicker border has been chosen. Lines indicate dependencies and associations, among variables identified by the “data driven” approach.]

From the network (**Figure 1**) we found that anxiety aspects of attachment styles were related to anxiety. The theory of attachment was originally formulated by Bowlby (1973) who conceptualized the human need to form affectional bonds with others and later Ainsworth et al. (1978) specified three attachment styles: secure, avoidant and anxious/ambivalent. Need for Approval and Preoccupation with Relationship of the Attachment Style Questionnaire (Feeney et al., 2004), are both subscales indicating the presence of an anxious attachment style. We found that these two scales were related to anxiety in our graphical model. Need for approval reflects the need for acceptance and confirmation from others. It corresponds to the fearful and preoccupied attachment styles described by Bartholomew and Horowitz (1991). Preoccupation with Relationship refers to the anxious and dependent tendency of acting in relationships, which constitutes a key characteristic of anxious and ambivalent attachment (Hazan and Shaver, 1987). Studies have shown that an insecure attachment style is associated to anxiety (Eng et al., 2001) and this attachment style can predict anxiety disorders (Marganska et al., 2013). From the graphical model, we hypothesize an indirect link between anxious attachment style and intention to breastfeed.

Our study should be interpreted in light of several limitations. It should be noted that non-Italian women and those under 18 years of age were excluded from the study. Caution in generalizing these results is therefore warranted. Furthermore, another limit of our study is that there was no verification of actual breastfeeding rates at a second time-point, as it was a cross-sectional study measuring solely breastfeeding propensity. However, our study is the first to investigate both socio-demographic and psychological variables that affect breastfeeding intentions in Italian women. We are aware that that the decision-making process involved in breastfeeding is multifactorial; however, our study focused mainly on demographic and psychological variables and we did not therefore take into consideration cultural, political, or physiological variables. Our aim was to empirically support clinical observations and give health workers information that could guide their contact with every pregnant woman individually. Knowing that anxiety and Neuroticism can negatively affect breastfeeding intention allows the obstetrician and the gynecologist to give specialized care to women who present these characteristics.

In the future it would be interesting to compare actual breastfeeding rates in a similar sample of women to see how faithful women remain to their intentions. Furthermore, it would be interesting to see if personality plays a role in long-term breastfeeding choice. We could also compare samples in different Italian hospitals and perhaps compare breastfeeding rates between our hospital in the north of Italy and other hospitals in other regions.

## Conclusion

We suggest that women with lower intentions to breastfeed could be identified early on during pregnancy. Our study helped to identify psychological and demographic variables, which serve as barriers to the recommended breastfeeding rates in Italy. Considering the results of this study, healthcare workers should pay special attention and screen for both psychological variables, that is, Neuroticism and anxiety and socio-demographic variables, namely employment, age and type of feeding received as a child. Psychological variables are, in particular, seen as potential intervention targets. Monitoring at-risk future mothers during the antenatal period gives the opportunity to improve breastfeeding intentions by, for example, trying to reduce anxiety levels in pregnant women.

## Ethics Statement

The study was approved by the Hospital Medical Ethical Committee (IRCCS San Raffaele Hospital). A written informed consent was obtained from all the participants at the time of questionnaire completion. Only women who agreed voluntarily to take part in the research were included.

## Author Contributions

VDM was responsible for the conception and design of the research project and approving the final copy. LC was responsible for drafting the work, revising it critically, drafting the study design and she was also involved in data acquisition. MB and CJ were responsible for drafting the work, revising it, and conducting research of the intellectual content. FC and CB were responsible for statistical analyses and data interpretation. AO and SR were responsible for data collection and data interpretation. MC and LS were responsible for the conception and design of the research project and approving the final copy. All authors are thus accountable for all aspects of the work, also with respect to the integrity and accuracy of it.

## Conflict of Interest Statement

The authors declare that the research was conducted in the absence of any commercial or financial relationships that could be construed as a potential conflict of interest.
